# Assessment of Sulfur Deficiency under Field Conditions by Single Measurements of Sulfur, Chloride and Phosphorus in Mature Leaves

**DOI:** 10.3390/plants7020037

**Published:** 2018-04-28

**Authors:** Philippe Etienne, Elise Sorin, Anne Maillard, Karine Gallardo, Mustapha Arkoun, Jérôme Guerrand, Florence Cruz, Jean-Claude Yvin, Alain Ourry

**Affiliations:** 1 Normandie Université, 14032 Caen CEDEX 5, France; elisesorin@live.fr (E.S.); Anne.Maillard@roullier.com (A.M.); alain.ourry@unicaen.fr (A.O.); 2 Etienne Philippe, Normandie Université-INRA 950 Ecophysiologie Végétale, Agronomie et nutritions N, C, S, Esplanade de la Paix, UNICAEN, CS14032, 14032 Caen CEDEX 5, France; 3 INRA, UMR 950 Ecophysiologie Végétale, Agronomie et nutritions N, C, S, Esplanade de la Paix, UNICAEN, CS14032, 14032 Caen CEDEX 5, France; 4 Centre Mondial d’Innovation, CMI, Groupe Roullier, 27 Avenue Franklin Roosevelt, 35400 Saint-Malo, France; Mustapha.Arkoun@roullier.com (M.A.); fcruz@roullier.com (F.C.); jcyvin@roullier.com (J.-C.Y.); 5 UMR Agroécologie, AgroSup Dijon, INRA, Université Bourgogne Franche-Comté, F-21000 Dijon, France; karine.gallardo-guerrero@inra.fr; 6 Vegenov BBV, Penn ar Prat, 29250 Saint Pol de Léon, France; jeromeguerrand@hotmail.com

**Keywords:** *Brassica napus*, diagnostic tools, indicator of S nutrition, S fertilization, *Triticum aestivum*, *Zea mays*

## Abstract

Determination of S status is very important to detect S deficiency and prevent losses of yield and seed quality. The aim of this study was to investigate the possibility of using the ([Cl^−^]+[NO_3_^−^]+[PO_4_^3−^]):[SO_4_^2−^] ratio as an indicator of S nutrition under field conditions in *Brassica napus* and whether this could be applied to other species. Different S and nitrogen (N) fertilizations were applied on a S deficient field of oilseed rape to harvest mature leaves and analyze their anion and element contents in order to evaluate a new S nutrition indicator and useful threshold values. Large sets of commercial varieties were then used to test S deficiency scenarios. As main results, this study shown that, under field conditions, leaf ([Cl^−^]+[NO_3_^−^]+[PO_4_^3−^]):[SO_4_^2−^] ratio was increased by lowering S fertilization, indicating S deficiency. The usefulness of this ratio was also found for other species grown under controlled conditions and it could be simplified by using the elemental ([Cl]+[P]):[S] ratio. Threshold values were determined and used for the clustering of commercial varieties within three groups: S deficient, at risk of S deficiency and S sufficient. The ([Cl]+[P]):[S] ratio quantified under field conditions, can be used as an early and accurate diagnostic tool to manage S fertilization.

## 1. Introduction

Sulfur deficiency in crops has been a major concern at the global scale for a number of years, and especially in crops that require higher levels of sulfur (S) than other cultivated species, such as oilseed rape. Sulfur limitation in oilseed rape crops provokes multiple changes in plant physiology leading to losses of yield and seed quality through modified lipid and protein compositions [1,2,3,4,5]. Sulfur deficiency in crops has increasingly been observed over the last 50 years [3,6,7,8]. The limited S availability in soil can be explained by several factors: significant reductions in S emission from industrial sources, use of mineral fertilizers without S, decreases in use of organic fertilizers, and changes in cropping systems including the use of high yielding commercial coupled with intensive management practices [3,9]. Seed yield from oilseed rape is usually improved by S fertilization, with doses depending on the multiple environment factors under which the crop is being grown [9]. Consequently, a precise recommendation for S fertilization is difficult, resulting in a wide variation in S doses applied under production conditions from 0 to 112 kg·S·ha^−1^ [10]. Consequently, S indicators are needed for an appropriate use of fertilizers and to avoid cases of excessive fertilization having environmental and economic consequences, and particularly to avoid cases of S deficiency so as to prevent losses of yield and seed quality [6,7].

The visual symptoms of severe S deficiency are characterized by general yellowing and purpling of leaves, which can be quantified by reflectance measurements. However, these visual symptoms of S deficiency can be confused with other deficiencies like N deficiency [6,11] and can be also imperceptible during moderated S deficiency which can decrease seed quality [12]. In order to diagnose S status in crops, methods derived from soil testing, modeling or plant analysis have already been proposed. Different soil tests have been developed to evaluate the amounts of inorganic SO_4_^2−^ in soil and the potential mineralization capacity of soil organic S compounds [13]. However, chemical analyses of soil have not proved successful to evaluate the amount of S available for the crops. Indeed, from these analyses, it’s difficult to predict the requirement of S fertilization because S availability in soils is highly variable due to fluctuations of mineralization, immobilization or leaching throughout the growing season [14]. A previous study has proposed a computer model to predict the risk of S deficiency in cereals in Great Britain using soil, atmospheric S deposition, and meteorological data [15]. This modeling approach identifies where S deficiency is likely to occur for a large scale assessment, but it is not designed to be used at the field scale [6]. In oilseed rape, a model based on the process of S allocation and partitioning might be relevant for determining the response to S nutrition under controlled conditions [16] but it needs to be validated under field conditions. 

Plant analyses appear to be a better method for identifying S deficiency and evaluate soil fertility and crop management strategies [17]. Different indicators of S nutrition using plant analysis have been proposed, i.e., the N:S ratio [18], the SO_4_^2−^:total S ratio ([19], SO_4_^2−^[20]), total S (Pinkerton 1998 [21], the malate:SO_4_^2−^ ratio [22], the molybdenum:S ratio [23],glutathione [24], amides [24], or expression of sulfur deficiency responsive genes[25,26], each presenting the advantages and drawbacks. Gene expression has potential as a sensitive indicator of S nutritional status because of its very early response of plant to S deficiency. Indeed, Howarth et al. [25] have shown that the expression of the *sdi1* gene was induced especially in leaf and root tissues in response to S deficiency in wheat. In oilseed rape, the up-regulation of genes encoding SO_4_^2−^transporters (*BnaSultr1;1*, *BnaSultr1;2, BnaSultr4;1* and *BnaSultr4;2*) is one of the first responses to S deprivation [26]. However, none of these molecular indicators can be easily used under field conditions as they require laboratory analysis and the use of control plants. The use of biochemical indicators in response to S deficiency has been suggested, such as a rapid decrease in glutathione [22,24,27,28] and a large accumulation of asparagine and glutamine [24]. However, the concentrations of these molecules can be influenced by factors other than S nutrition, such as salinity, water or temperature stress [29,30]) and their diagnostic usefulness has been questioned. Use of SO_4_^2−^and total S concentrations have been proposed by Scaife and Burns [20] and Pinkerton [21] as the most satisfactory indices of plant S status because of the very wide range of values with clear changes from deficiency to sufficiency. These parameters present the added advantage of requiring only one measurement.

It has been shown that ratios of mineral (or organic) plant compounds can predict S nutrition more reliably than absolute values of one or more contents of mineral (or organic) compounds. Indeed, it has been suggested that ratios between compounds varied less with plant growth stages and growth rates than absolute values [7,17,22,31]. The SO_4_^2−^:total S ratio has been proposed by Spencer and Freney [19] as a useful guide to S fertilization because it is not strongly affected by the age of the plant or N supply. However, Scaife and Burns [20] criticized this use of the SO_4_^2−^:total S ratio instead of SO_4_^2−^or total S alone because the change in total S is partly due to the levels of SO_4_^2−^, thus the numerator (SO_4_^2−^) depends on the denominator (total S), which reduces the sensitivity of the indicator. Rasmussen et al. [18] proposed the N:S ratio for wheat at the vegetative stage. Compared to the total S concentration of vegetative tissues, the N:S ratio also decreases, but to a lesser extent, so that its use as an indicator requires sampling at a precise growth stage [2]. Moreover, this ratio should be interpreted with caution because it can be affected by oversupply of N without S deficiency. Recently, the N:S ratio in grain has been used as the most accurate diagnostic tool by many agronomists and the farming industry for S deficiency in crops. However, the grain N:S ratio is not a predictive test as it indicates that the harvested crop may have been S deficient, so that S fertilization can be increased for the following crop [32,33]. Alternately, Blake-Kalff et al. [22,32] proposed the malate: SO_4_^2−^ ratio as a diagnostic indicator of S deficiency in wheat and oilseed rape. This ratio is based on the inverse relationship between malate and SO_4_^2−^concentrations in leaves; when the SO_4_^2−^concentrations decrease, the malate concentrations increase, and vice versa. This ratio has the advantage of being stable throughout plant development when the S supply is sufficient. Moreover, it can be calculated from a single analytical run by using the peak areas of malate and SO_4_^2−^, avoiding the problems of accurate calibration. Studies on the reliability of the malate:SO_4_^2−^ ratio indicate that it overestimated diagnosis of S deficiency and suggested that threshold values need to be reconsidered [33,34]). More recently, Maillard et al. [23] have demonstrated that the [Mo]/[S] constitutes an accurate indicator to diagnose S deficiency. However, because the range of Mo leaf contents are usually low, measurements of this element need complex and senesitive laboratory methods (such as ICPMS-HR) which make difficult to use routinely this diagnostic tool. Finally, Sorin et al. [26] proposed that the ([Cl^−^]+[NO_3_^−^]+[PO_4_^3−^]):[SO_4_^2−^]ratio could provide a relevant early indicator of S deficiency in oilseed rape. This new early indicator of S nutrition identified under controlled conditions in oilseed rape is based on early physiological regulation that occurs before significant metabolic perturbations. It was shown that vacuolar SO_4_^2−^, acting as an osmoticum, was efficiently remobilized during S deprivation to sustain plant growth. This was compensated osmotically by a vacuolar accumulation of Cl^−^, NO_3_^−^ and PO_4_^3−^. This mechanism of osmotic compensation occurred as early as 3 days after S deprivation and long before metabolic disturbances and growth reduction in oilseed rape.

The main objectives of the current work were to validate the use of the ([Cl^−^]+[NO_3_^−^]+[PO_4_^3−^]):[SO_4_^2−^] ratio as an indicator of S nutrition under field conditions. The first objective was to test the ([Cl^−^]+[NO_3_^−^]+[PO_4_^3−^]):[SO_4_^2−^] ratio in leaves of oilseed rape grown under field conditions with different S and N fertilization rates on an S deficient field, and consequently if it constitutes a relevant indicator to predict the risk of S deficiency under field conditions. The second objective was to test the genericity of the ([Cl^−^]+[NO_3_^−^]+[PO_4_^3−^]):[SO_4_^2−^] ratio as an indicator of S deficiency in other species of different families such as *Brassica oleracea*, *Triticum aestivum*, *Zea mays*, *Medicago truncatula* and *Solanum lycopersicum* under controlled conditions. Finally, the last aim was to simplify the quantification of the ([Cl^−^]+[NO_3_^−^]+[PO_4_^3−^]):[SO_4_^2−^] ratio, by using elemental analysis of Cl, P and S in order to facilitate its quick measurement on samples harvested in field and allow an adjustment of S fertilization in short time delay after the diagnostic of S deficiency.

## 2. Materials and Methods

### 2.1. Field Experiments and Plant Sampling

#### 2.1.1. Field Experiment 1: Study of Different Fertilization Rates on an S-Deficient Field

The experimental site was selected from a previous study [10] showing an S deficiency in *Brassica napus* L. grown in 2009. This field of 11.3 ha was located at Ondefontaine, France (48°59′18.69” N, 00°41′56.70” W). A winter oilseed rape variety (*Brassica napus* L., 95% cv DK Exstorm and 5% cv Alicia) was sown with a density of 35–40 plants m^−^² on 27 august 2013. The previous crop was spring barley (*Hordeum vulgare* L.). Chemical analyses of the loam soil provided an S content of 220 mg kg^−1^ and a pH value of 6.4. Before crop establishment, organic fertilizer (40 m^3^ ha^−1^ of bovine manure) was applied on the whole field corresponding to 122 kg N ha^−1^ and 44 kg S ha^−1^. The field was separated into seven randomized plots fertilized with different doses of N and S mineral fertilizer. One plot was unfertilized. Other plots were fertilized on 27 February 2014 with three different doses of S fertilizer: 0, 12, and 36 kg S ha^−1^assulfuric anhydride (36% SO_3_) and ammonium nitrate to obtain 65 kg N ha^−1^on each plot. Then, on 27 March 2014, a second N fertilization with 60 kg N ha^−1^ (liquid N containing 50% urea, 25% ammonium N and 25% nitrate N) was applied to three plots that thus received 125 kg N ha^−1^ after two fertilizer applications.

Five harvests were performed between January and July 2014: before fertilization (at the rosette stage and at the start of stem elongation (GS30), 30 January 2014 and 12February 2014), 15 days after S fertilization (start of the visible bud stage (GS50), 14March 2014), 47 days after S fertilization (stage of pods formation (GS70), 15 April 2014) and finally after 60 days (stage of pods formation (GS70), 28 April 2014). Two batches of leaves, mature leaves (from the lower canopy) and young leaves (from the higher canopy), were harvested using 20 leaves randomly collected from each plot and with three replicates. Leaves were freeze-dried for dry weight (DW) determination and ground, using an oscillating grinder (MM400, Retsch, Haan, Germany) to fine powder for further analysis. 

#### 2.1.2. Field Experiment 2: Study of 45 Commercial Varieties before Fertilization and Flowering

Forty-five commercial varieties of oilseed rape were selected in France according to different locations (Supplemental data SD1), different agricultural practices (dose of fertilizers, previous crop, tillage), and under contrasting soil and climate conditions that may affect SO_4_^2−^leaching and hence SO_4_^2−^availability. The farmers, identified with the help of DATAGRI (Lyon, France), collaborated in the present study. Plots have been numbered from 1′ to 45′ according to the increasing ([Cl^−^]+[NO_3_^−^]+[PO_4_^3−^]):[SO_4_^2−^] ratio. Two batches of leaves comprising mature leaves (from the lower canopy) and young leaves (from the higher canopy), each of them consisting of 20 leaves, were randomly collected from each of the 45 fields in February 2014, just at the end of winter corresponding to the start of stem elongation (GS30) and before fertilization. Leaves were freeze-dried before laboratory processing.

In addition, four commercial varieties were studied before and after fertilization. Agricultural practices were managed by the farmers and the authors did not intervene in the choice of fertilizer doses. These four plots were located in France at Indre (N 46°36′48″, E 02°06′41″)corresponding to plot number 8′, Loiret (N 48°06′59″, E 02°25′04″) corresponding to plot number 11′, Indre-et-Loire (N 47°06′05″, E 00°22′14″) corresponding to plot number 20′ and Seine-et-Marne (N 48°30′28″, E 02°49′56″) corresponding to plot number 36′. Two harvests occurred between January and May 2014: before fertilization (at the rosette stage and at the start of stem elongation (GS30), February 2014) and after N and S fertilizers were applied by the farmers (stage of pods formation (GS70), April 2014). Leaves were collected as for the 45 plots, with three replicates of leaves collected.

#### 2.1.3. Field Experiment 3: Study of 56 Commercial Varieties after Fertilization and Flowering

In order to maximize variability for S status at the late stage of oilseed rape development, 56 commercial varieties were selected in Calvados (Lower Normandy, France) during a previous study [10]. The numbering of plots was performed in accordance with Sarda et al. [10]. Fifty senescent leaves (i.e., yellowing, just before their abscission) were randomly collected after flowering (May 2009) from each of the 56 fields. Four biological replicates of plants were used per condition and harvesting date. Leaf samples were kept at 4 °C for a short period before drying at 60 °C for 72 h.

### 2.2. Multispecies Experiment under Controlled Conditions

In order to assess if data obtained with *B. napus* could be extrapolated to other species, experiments using *B. oleracea*, *T. aestivum*, *Z. mays*, *S. lycopersicum, M, truncatula* and *B. napus* grown under controlled conditions were conducted under different culture conditions described in Supplemental data SD2 with optimal and sub-optimal S fertilization. Leaves were harvested and frozen immediately in liquid nitrogen and stored at −80 °C until freeze-drying for further analysis.

### 2.3. SO_4_^2−^, PO_4_^3−^, Cl^−^ and NO_3_^−^Analysis

Ions were extracted from 30 mg of freeze-dried plant material and were initially mixed with 1.5 mL of 50% ethanol solution. After incubation at 40 °C for 1 h, the extract was centrifuged at 12,000× *g* for 20 min and the supernatant was collected. This step was repeated on the pellet and the resulting supernatant obtained was pooled with the previous one. All these operations (i.e., incubation and centrifugation) were repeated twice, but with 1.5 mL of ultra-pure water and incubation at 95 °C. All the supernatants were pooled and evaporated under vacuum (Concentrator Evaporator RC 10.22, Jouan, Saint-Herblain, France). The dry residue was re-suspended in 1.5 mL of ultra-pure water and was filtered on 45 µm filters.

Thereafter, anion contents were determined using high performance liquid chromatography (HPLC) with a conductivity detector (ICS3000, Thermo Scientific-Dionex, Villebon-sur-Yvette, France). The eluent solution for anion analysis consisted of 4.05 mM Na_2_CO_3_ and 1.26 mM NaHCO_3_ and was pumped isocratically over an analytical column (AS22 4 × 250 mm).

### 2.4. S, P, Cl and N Analysis

After drying and grinding, leaf samples were placed sample cups and S, P, and Cl were determined with X-ray fluorescence (XRF) analysis (Portable XRF S1 TITAN 800, Bruker, Kalkar, Germany). Quantification of each element was performed using calibration curves of samples quantified by High Resolution Inductively Coupled Plasma Mass Spectrometry (HR ICP-MS, Thermo Scientific, Element 2^TM^, Bremen, Germany) [35]. For N analysis, an aliquot of each dried sample was placed into tin capsules using a microbalance and the total N content was determined with a continuous flow isotope ratio mass spectrometer (Horizon, NU Instruments, Wrexham, UK) linked to a C/N/S analyzer (EA3000, Euro Vector, Milan, Italy).

## 3. Statistical Analysis

For field experiment 1, three replicates corresponding to a pool of 20 leaves of 20 independent plants were collected. Data are reported as mean ± SE for *n* = 3 and were analyzed by Student’s test (Excel software, Microsoft Corporation, Redmond, Washington, DC, USA) and marked by different letters when they were significantly different between treatments at a given date, at *p* < 0.05. For the second part of field experiment 2, three replicates corresponding to a pool of 20 leaves of 20 independent plants were collected. These data are also reported as mean ± SE for *n* = 3 and were analyzed by Student’s test (Excel software) and marked by different letters when they were significantly different between plots at a given date, at *p* < 0.05, and marked by one or several asterisks when significantly different before and after fertilization. All experiments on different species in controlled conditions were conducted with four independent biological replicates. Data are reported as mean ± SE for *n* = 4. All data of the multispecies experiment were analyzed by Student’s test (Excel software) and marked by one or several asterisks when significantly different between controls and S-deprived plants.

## 4. Results

### 4.1. Under Field Conditions, a Decrease in the SO_4_^2−^ Content Was Compensated by an Increase in the (Cl^−^+NO_3_^−^+PO_4_^3−^) Contents in Oilseed Rape Leaves Leading to an Increase in the ([Cl^−^]+[NO_3_^−^]+[PO_4_^3−^]):[SO_4_^2−^]Ratio

**Figure 1:**
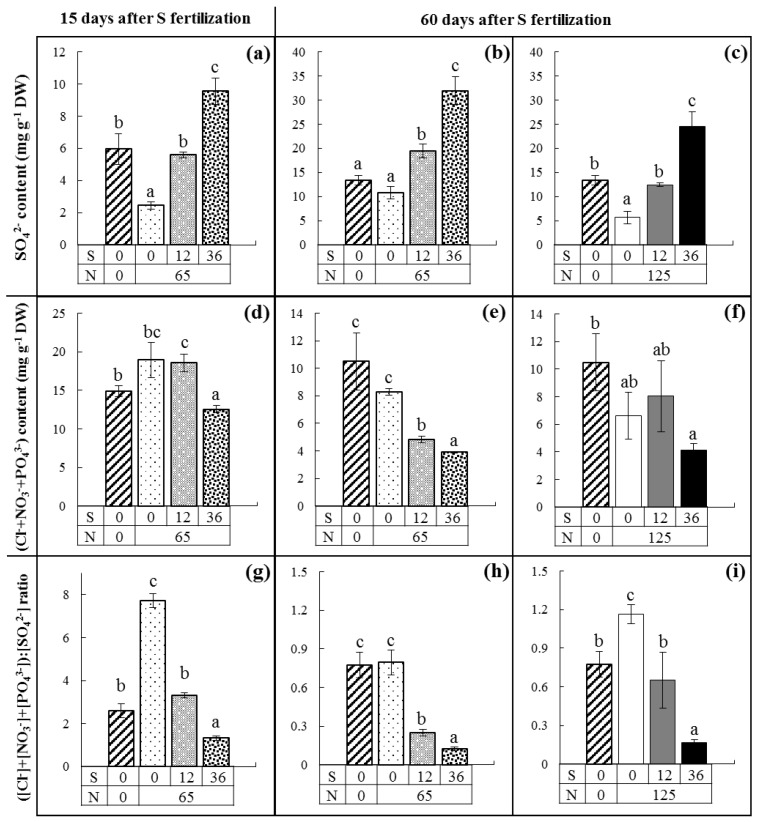
(**a**–**c**) SO_4_^2−^contents (mg g^−1^ DW); (**d**–**f**) (Cl^−^+NO_3_^−^+PO_4_^3−^) contents (mg g^−1^ DW) and (**g**–**i**) the ([Cl^−^]+[NO_3_^−^]+[PO_4_^3−^]):[SO_4_^2−^] ratio in mature leaves of oilseed rape grown under field conditions (field experiment 1), after (**a**,**d**,**g**) 15 and (**b**,**c**,**e**,**f**,**h**,**i**) 60 days of S fertilization. Plants received no mineral fertilization (hatched bars, 0 kg S ha^−1^, 0 kg N ha^−1^) or 0 (white bars), 12 (gray bars) or 36 (black bars) kg S ha^−1^, with 65 (dashed bars) or 125 (full bars) kg N ha^−1^. Within the same graph, letters when different indicate significant difference between fertilization treatments for *p* < 0.05.

For field experiment 1, SO_4_^2−^, Cl^−^, NO_3_^−^and PO_4_^3−^ contents and the ([Cl^−^]+[NO_3_^−^]+[PO_4_^3−^]):[SO_4_^2−^] ratio were quantified in leaves of oilseed rape at 15, 47, and 60 days after S fertilization. Similar observations were found at 47 and 60 days after S fertilization, and to simplify readings, only the results at 60 days after S fertilization are presented. The SO_4_^2−^content (Figure 1a–c) was decreased significantly in mature leaves by a reduction in S fertilization (from 36, 12, to 0 kg S ha^−1^), whatever the dose of N fertilization (65 or 125 kg N ha^−1^). The decrease in SO_4_^2−^ content in mature leaves was compensated by an increase in Cl^−^, NO_3_^−^, and PO_4_^3−^ contents (Figure 1d–f). The lowest (Cl^−^+NO_3_^−^+PO_4_^3−^) content was found in mature leaves of plants fertilized with the highest dose of S (i.e., 36 kg S ha^−1^). The ([Cl^−^]+[NO_3_^−^]+[PO_4_^3−^]):[SO_4_^2−^]ratio (Figure 1g–i) differentiated the three plots according to S fertilization: 0, 12, 36 kg S ha^−1^. Mature leaves of plants receiving N fertilization (65 or 125 kg N ha^−1^) without S had the highest ([Cl^−^]+[NO_3_^−^]+[PO_4_^3−^]):[SO_4_^2−^]ratio, whereas the mature leaves of plants with 36 kg S ha^−1^ had the lowest, and mature leaves of plants with 12 kg S ha^−1^ presented an intermediate ([Cl^−^]+[NO_3_^−^]+[PO_4_^3−^]):[SO_4_^2−^]ratio.

Interactions between N and S fertilization were also found. It can be assumed that the growth rate was mostly reduced by low N availability. For example, 15 days after S fertilization, the biomass of mature leaves was 5.03 ± 0.38 g DW leaf^−1^ in the unfertilized plot whereas in the plot with 65 kg N ha^−1^ the biomass was 6.70 ± 0.01 g DW leaf^−1^ (*p* < 0.001). This reduced growth of N unfertilized plants had a direct consequence on the SO_4_^2−^ content because SO_4_^2−^ requirements were lower than in N fertilized plants, which used SO_4_^2−^ to ensure growth. For example, for the same S fertilization rate, the SO_4_^2−^ content (Figure 1a–c) in mature leaves of plants without N fertilization was significantly higher than (Figure 1a,c) or similar to (Figure 1b) the content in leaves of plants with N fertilization. Moreover, compared to leaves from plants with N fertilization and without S fertilization, the Cl^−^+NO_3_^−^+PO_4_^3−^ content (Figure 1d–f) in mature leaves of the N unfertilized plot was unchanged after 15 and 60 days. As a consequence, the ([Cl^−^]+[NO_3_^−^]+[PO_4_^3−^]):[SO_4_^2−^] ratio (Figure 1g–i) decreased significantly (Figure 1g,i) or was similar (Figure 1h) in the absence of N mineral fertilization in mature leaves, reflecting lower S requirements. In the same way, plant growth was stimulated by a second N fertilization of 60 kg N ha^−1^ (corresponding to plots with 125 kg N ha^−1^) and consequently the S requirements were increased, reducing the SO_4_^2−^ content in mature leaves (*p* < 0.05, when comparing Figure 1b,c). The second N fertilization had no significant impact on the (Cl^−^+NO_3_^−^+PO_4_^3−^) contents of mature leaves (Figure 1f) but these ions tended to increase for a given dose of S fertilization (0, 12 or 36 kg S ha^−1^) compared to plants with 65 kg N ha^−1^ (Figure 1e). So, when comparing Figure 1h and i, the ([Cl^−^]+[NO_3_^−^]+[PO_4_^3−^]):[SO_4_^2−^] ratio was increased in mature leaves of plants receiving high N fertilization (Figure 1i), which reflected a higher requirement for S. 

The same analyses were also performed with younger leaves (data not shown), which showed a similar trend to mature leaves but with a lower amplitude of response of the ([Cl^−^]+[NO_3_^−^]+[PO_4_^3−^]):[SO_4_^2−^] ratio between plants receiving N or S fertilizations. Consequently, for the other experiments only data obtained from mature leaves will be given.

### 4.2. The ([Cl^−^]+[NO_3_^−^]+[PO_4_^3−^]):[SO_4_^2−^]Ratio Can Be Used in Other Plant Species to Detect S Deficiency under Controlled Conditions

The ([Cl^−^]+[NO_3_^−^]+[PO_4_^3−^]):[SO_4_^2−^] ratio was also calculated in leaves of *B. oleracea*, *T. aestivum*, *Z. mays*, *M. truncatula*, *S. lycopersicum*, and *B. napus* grown under controlled conditions with or without S restriction (Table 1). In all species, S deficiency decreased the SO_4_^2−^ content in leaf tissue and significantly increased (Cl^−^+NO_3_^−^+PO_4_^3−^) contents. Consequently, the ([Cl^−^]+[NO_3_^−^]+[PO_4_^3−^]):[SO_4_^2−^] ratio was highly and significantly increased by S deficiency as soon as threedays after treatment in *B. napus*, eight days in *T. aestivum*, five days in *Z. mays*, and eight days in *M. truncatula*, and this increase was even amplified in the mid- to long-term. However, if no early harvest was performed with *B. oleracea* and *S. lycopersicum*, a significant increase in the ([Cl^−^]+[NO_3_^−^]+[PO_4_^3−^]):[SO_4_^2−^] ratio during S deficiency was found in the longer term (i.e., after 135 days of S deficiency in *B. oleracea* and after 75 days in *S. lycopersicum*). Finally, it must be pointed out that the absolute value of this ratio was species specific, and probably a result of the intrinsic capacity of a plant species to store SO_4_^2−^ in leaves, as illustrated by the two *Brassica* species as well as tomato which accumulated higher levels of SO_4_^2−^ (Table 1).

### 4.3. The Simplified ([Cl]+[P]):[S] Ratio Could Be Used for an Easier and Faster Determination of S Deficiency under Field Conditions

**Figure 2:**
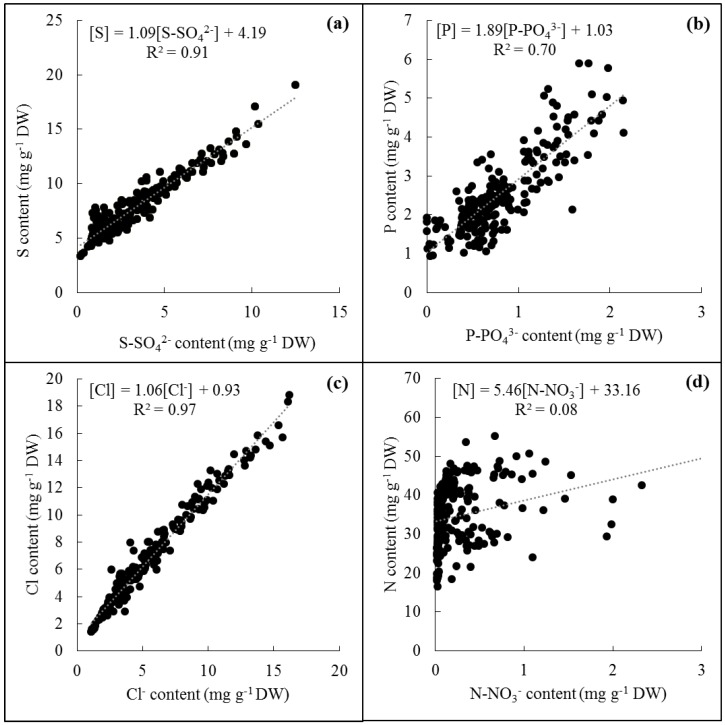
Correlation between (**a**) S-SO_4_^2−^ and S content (mg g^−1^ DW); (**b**) P-PO_4_^3−^ and P content (mg g^−1^ DW); (**c**) Cl^−^ and Cl content (mg g^−1^ DW) and (**d**) N-NO_3_^−^ and N content (mg g^−1^ DW). Ion contents were quantified by HPLC and element contents by X-ray fluorescence (XRF), except N which was quantified by IRMS, in leaves of oilseed rape grown under field conditions (field experiment 1 and the second part of field experiment 2). Data used were obtained from plants of all fertilization treatments.

**Figure 3:**
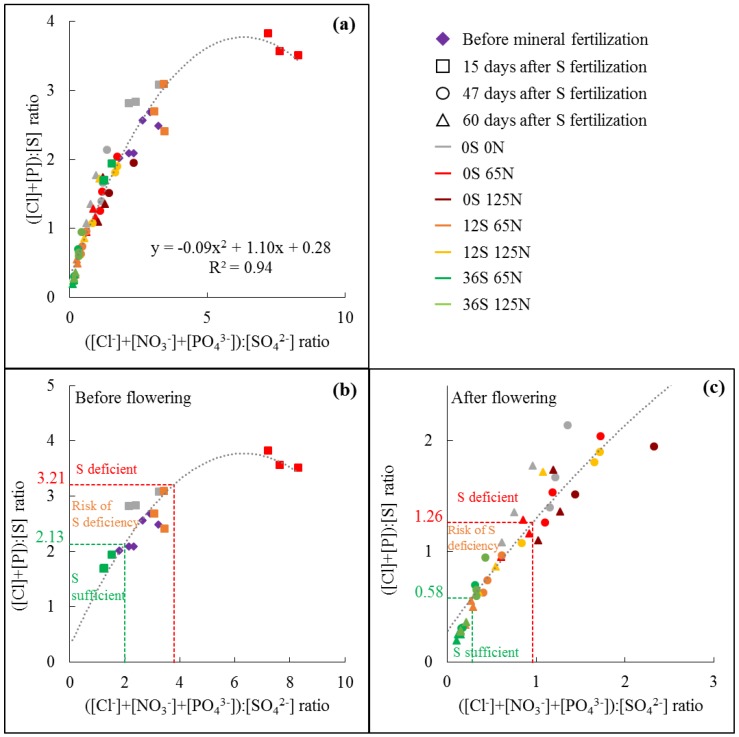
Correlation between the ([Cl^−^]+[NO_3_^−^]+[PO_4_^3−^]):[SO_4_^2−^] ratio and the ([Cl]+[P]):[S] ratio in mature leaves of oilseed rape submitted to different N and S fertilization rates (field experiment 1), (**a**) for all data points (all fertilization treatments, leaves harvested before mineral fertilization, or 15, 47 or 60 days after S fertilization), or for mature leaf samples harvested (**b**) before or (**c**) after flowering. Thresholds of the ([Cl]+[P]):[S] ratio were determined as the difference or sum between the mean and the 95% confidence interval of plants receiving 0 or 36 kg S ha^−1^, defining three groups of plants: S deficient, at risk of S deficiency and S sufficient plants.

The resulting correlations between ion contents measured by HPLC and element contents measured by handheld X-Ray Fluorescence spectrometry (XRF) are given in Figure 2. In oilseed rape leaves, the ions SO_4_^2−^, PO_4_^3−^, and Cl^−^were highly linearly correlated to their respective elements S, P, and Cl (Figure 2a–c) with correlation coefficients of 0.91, 0.70, and 0.97, respectively. Such strong linear relationships can be easily explained by the fact that these ions represented the main proportion of the corresponding element in mature leaves. A maximum (top of the curve) of 56%, 70%, and 95% of P, S and Cl were in the form of PO_4_^3−^, SO_4_^2−^, and Cl^−^, respectively. On the other hand, no significant correlation was found between NO_3_^−^ and N contents in leaves of oilseed rape (Figure 2d), which was the result of very low NO_3_^−^ contents in leaves of plants grown under field conditions (data not shown). Consequently, for plants from field experiment 1, the anionic ratio ([Cl^−^]+[NO_3_^−^]+[PO_4_^3−^]):[SO_4_^2^^−^] was significantly correlated to the ([Cl]+[P]):[S] ratio (Figure 3a) by a second order polynomial equation with a correlation coefficient of 0.94 showing a saturation plateau for higher values of the ([Cl]+[P]):[S] ratio. This previous graph was split in order to separate data from plants harvested before flowering (Figure 3b, plants harvested before fertilization and 15 days after fertilization when plants were at the start of the visible bud stage) and after flowering (Figure 3c, plants harvested 47 and 60 days after S fertilization for which plants were at the stage of pod formation) to assess whether this ratio was affected by plant development and then to determine potential threshold values. Before flowering (Figure 3b), both ratios were higher than after flowering (Figure 3c) with the values being between 1.69 and 3.83 for the ([Cl]+[P]):[S] ratio in plots with high S and low S fertilization, respectively. After flowering the ([Cl]+[P]):[S] ratio values (Figure 3d) were lower, between 0.19 (high S fertilization) and 2.14 (no S fertilization). Before any mineral fertilization, the ([Cl]+[P]):[S] ratios of leaves were between 2.02 and 2.68 (Figure 3a,b purple diamonds) and without S fertilization (i.e., 0 kg S ha^−1^, orange squares) the ratio increased and to a lesser extent for the unfertilized plot (i.e., 0 kg S ha^−1^ and 0 kg N ha^−1^, red squares), whereas it decreased at 36 kg S ha^−1^ (green squares).

### 4.4. Determination of Threshold Values of the ([Cl]+[P]):[S]Ratio and Their Use on Independent Fields

Two sets of threshold values of the ([Cl]+[P]):[S]ratio were determined: before flowering with a potential use to determine whether or not S fertilization was required and after flowering to assess whether plants were S deficient. Threshold values were then calculated from data given in Figure 3b and c as the sum or difference between the ([Cl]+[P]):[S]ratio mean and the 95% confidence interval of S sufficient plots (36 kg S ha^−1^) and S deficient plots (0 kg S ha^−1^), respectively. The threshold values were then 2.13 and 3.21 before flowering, whereas they were 0.58 and 1.26 after flowering. Such thresholds would allow clustering of the plots within three groups of plots: S sufficient (ratio <2.13 or 0.58 after flowering), at risk of S deficiency (ratio between 2.13 and 3.21 or between 0.58 and 1.26 after flowering), and S deficient (ratio >3.21 or 1.26 after flowering) (Figure 3b,c).

**Figure 4:**
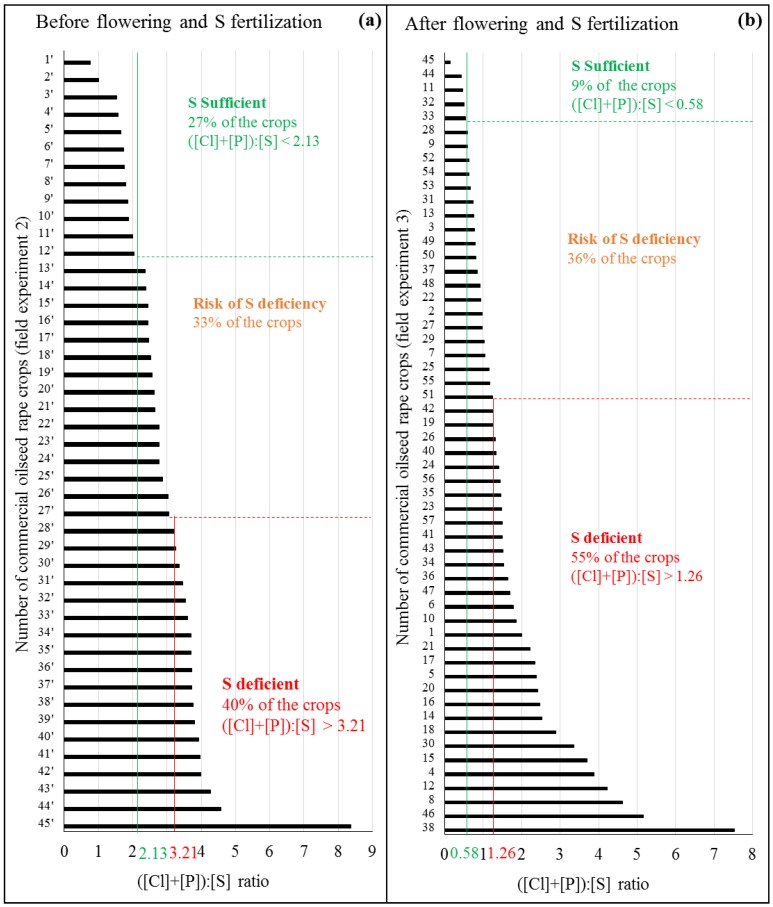
Commercial oilseed rape varieties (field experiments 2 and 3) classified according to decreasing values of the ([Cl]+[P]):[S] ratio in mature leaves quantified (**a**) before flowering and S fertilization (Field experiment 2 using 45 commercial varieties from different locations in France, see SD1) or (**b**) after flowering and S fertilization (Field experiment 3 using 56 commercial varietieslocalized in Calvados, Lower Normandy, France). Threshold values of the ([Cl]+[P]):[S] ratio classified these oilseed rape plots into three S status groups: S deficient, at risk of S deficiency and S sufficient plants.

This classification of oilseed rape plots into three groups, according to their S nutrition status evaluated by the leaf ([Cl]+[P]):[S]ratio, has been tested on two sets of independent commercial varieties(Figure 4). The first set corresponded to 45 commercial varieties from different locations in France and for which leaves were harvested before flowering and S fertilization (Figure 4a, field experiment 2). The second set corresponded to 56 other commercial varieties located in Calvados (Lower Normandy, France) and for which leaves were harvested after flowering and S fertilization (Figure 4b, field experiment 3). In the first case (Figure 4a), the use of the threshold values suggested that 27%, 33%, and 40% of the plots analyzed before flowering would be classified as S sufficient, at risk of S deficiency or as S deficient, respectively. After flowering (Figure 4b) 9%, 36%, and 55% of the analyzed plots fell into the previously cited groups.

In order to test if the ([Cl]+[P]):[S]ratio and associated thresholds take into account the availability of soil S and its interaction with the rate of S fertilization, the mature leaves, and the soil of four plots derived from field experiment 2 (i.e., plots # 8′, 11′, 20′, and 36′ represented in Figure 4a and supplemental data SD1) were harvested and analyzed before and after S fertilization (Table 2). These four oilseed rape plots were then classified within three groups (from S deficient to S sufficient) according to the ([Cl]+[P]):[S]ratio, determined before and after S fertilization. It was found that before flowering and S fertilization, the lower the S content in the soil, the higher the ([Cl]+[P]):[S]ratio and vice versa, which validated the possibility of using the([Cl]+[P]):[S]ratio as an indicator of sulfur status (Table 2). After S fertilization (from 0 to 100kg S ha^−1^), the ([Cl]+[P]):[S]ratio was decreased partly due to plant development (see plot # 11′ without S fertilization in Table 2 as well as data of experiment 1 Figure 3a,c) but mostly as a result of the S fertilization rate. For example, plot # 20′, classified at risk of S deficiency before S fertilization, received 100 kg S ha^−1^ which decreased its ([Cl]+[P]):[S]ratio by 78% (from 2.64 to 0.59) and thus ascribed it to the sufficient S availability group. Overall, the simple use of the leaf ([Cl]+[P]):[S]ratio before fertilization on these four plots indicated that plots # 8′ and 11′ would not have required any S fertilization. Nevertheless, the decrease in this ratio after flowering suggested that these plots were S deficient with little or no S fertilization. While plots # 36′ and 20′ seemed to require S fertilization, plot # 36′ would have required a higher dose of S, and plot # 20′ would have required a dose of S probably below those that were actually applied.

## 5. Discussion

As previously reported, oilseed rape requires fairly high levels of S in order to maintain yield and seed quality [2,5,14]. Since a general S deficiency in cultivated soils has been described [3,6,7,8], S fertilization is usually provided to oilseed rape, but less frequently on other crops. Moreover, the dose used or recommended does not take into account the real needs of the plants and growth potential. This can lead to excessive or insufficient S fertilization [10]. Hence, the development of new diagnostic tools of S deficiency, usable under field conditions, is required. The aims of this work were to identify and validate a diagnostic tool derived from early physiological processes occurring during oilseed rape adaptations to S deficiency.

### 5.1. The ([Cl^−^]+[NO_3_^−^]+[PO_4_^3−^]):[SO_4_^2−^] Ratio as an Indicator of S Nutrition under Field Conditions

Under field conditions, the highest Cl^−^, NO_3_^−^, PO_4_^3−^contents were found in mature leaves of plants with the lowest S fertilizations having the lowest SO_4_^2−^content, and hence the highest ([Cl^−^]+[NO_3_^−^]+[PO_4_^3−^]):[SO_4_^2−^] ratio (Figure 1a–i). These higher contents of Cl^−^, NO_3_^−^ and PO_4_^3−^ might help to maintain the osmotic potential when SO_4_^2−^is mobilized from the vacuole of mature leaves (Figure 1d–f). It was also found that leaves of plants grown under field conditions have accumulated Cl^−^ with the same order of magnitude that was previously found under hydroponic conditions. Indeed, oilseed rape leaves of field grown plants accumulated anions in the same range of concentrations found under hydroponic conditions: 2–16 mg Cl^−^ g^−1^ DW (4–20 mg Cl^−^ g^−1^ DW in hydroponic culture), 0.1–3 mg PO_4_^3−^ g^−1^ DW (2–7 mg PO_4_^3−^ g^−1^ DW in hydroponic culture) and 1–37 mg SO_4_^2−^ g^−1^ DW (2–42 mg SO_4_^2−^ g^−1^ DW in hydroponic culture). Only NO_3_^−^was found to accumulate in leaves at a much lower level under field conditions (0.1–5 mg *versus* 40–70 NO_3_^−^ g^−1^ DW under hydroponic culture). The same range of NO_3_^−^ contents in mature leaves has been previously reported by Blake-Kalff et al. [22] and Sarda et al. [10] in oilseed rape grown under field conditions. Nevertheless, the ([Cl^−^]+[NO_3_^−^]+[PO_4_^3−^]):[SO_4_^2−^] ratio was able to significantly differentiate plants receiving different doses of S fertilization under field conditions as soon as 15 days after S supply (Figure 1g–i). This ratio was also found to be sensitive to N fertilization. Janzen and Bettany [1] and Zhao et al. [36] reported that higher N fertilization increased plant growth rates and consequently S requirements and hence may aggravate the S deficiency of oilseed rape plants.

Whatever the plant species, the leaf ([Cl^−^]+[NO_3_^−^]+[PO_4_^3−^]):[SO_4_^2−^] ratio was significantly and precociously increased by reduced SO_4_^2−^ availability in nutrient solutions, resulting from a disappearance in SO_4_^2−^in leaves that was accompanied by an increase in (Cl^−^+NO_3_^−^+PO_4_^3−^)content. These results strongly suggest that this ratio could be used as a diagnostic tool for a wide range of cultivated plant species.

### 5.2. Using the ([Cl]+[P]):[S] Ratio Instead of the ([Cl^−^]+[NO_3_^−^]+[PO_4_^3−^]):[SO_4_^2−^] Ratio

Because Cl^−^, NO_3_^−^, PO_4_^3−,^ and SO_4_^2−^ cannot be quantified with enough accuracy with simple methods such as colorimetric test strips, another aim of this study was to find a way to simplify the analysis of this anionic ratio. Due to their consequent accumulation in their mineral form and the resulting strong linear correlations between anions and elements (Figure 2a–c), quantification of elements such as Cl, P, or S can provide an easy way to determine accurately the Cl^−^, PO_4_^3−^, and SO_4_^2^ contents in leaf tissues. As NO_3_^−^was not massively accumulated in leaves of plants grown under field conditions [10,22], we found that the ([Cl^−^]+[NO_3_^−^]+[PO_4_^3−^]):[SO_4_^2−^] and ([Cl]+[P]):[S]ratios were highly correlated by a second-degree polynomial equation (Figure 3a). This ([Cl]+[P]):[S]ratio was measured accurately by handheld XRF spectrometry, directly from ground dry leaf samples, which could be done from leaf samples harvested in the field. Another advantage of quantifying elements instead of anions relies on the fact that the plant samples can be stored for several hours or days after harvest without affecting elementary quantification, which will not be the case with anions.

### 5.3. Potential Thresholds of the ([Cl]+[P]):[S] Ratio

A successful diagnostic indicator as defined by Blake-Kalff et al. [22] needs to show a large response to different S fertilizations, remain stable during development, and be relatively easy to measure with little effort and with great accuracy. Most of these criteria are respected by the ([Cl]+[P]):[S] indicator. The precaution to be taken with this indicator is the use of threshold values depending on the developmental stage. According to previous study [17], threshold values should never be considered as absolute, but as a representation of a possible range resulting from uncontrolled or unknown variables. Having an index of nutrition with constant threshold values at different growing stages has already been questioned [6,20]. In this study, two threshold values for the two groups of development stages have been determined, allowing classification of plots into three S status groups: S deficient, at risk of S deficiency, and S sufficient, with the additional intermediate class taking into account the area of uncertainty. The survey of the S status of 45 commercial oilseed rape varieties in France, field experiment 2, has enabled mapping of their locations according to S requirements (Supplemental data SD1). The plots located in the north-east of France, which corresponds to the cereal plain, was mostly S sufficient. Other areas were more subject to S deficiency. Previous studies have shown that in response to a moderate S deficiency or a decrease in S availability during late stage of reproductive development, oilseed rape is able to maintain its growth and its yield components by increasing remobilization of previously stored S (especially SO_4_^2−^) in leaves [37,38]. This was probably the case for plots # 8′ and # 11′ (Table 2), which were classified as S sufficient before flowering and with little or no S fertilization, whereas they were classified as S deficient after flowering. The example of these two plots also suggests that the determination of soil S content is not reliable on its own to predict the plant S status, and which highlights the need for indicators at the plant level.

## 6. Conclusions

This study firstly showed that the accumulation of anions such as Cl^−^, NO_3_^−^, and PO_4_^3−^ in response to SO_4_^2−^remobilization triggered by S deficiency also occurred in oilseed rape grown under field conditions as previously shown in hydroponically grown plants. The same process was also found in species of different families. The derived leaf elementary ratio, ([Cl]+[P]):[S], seems to be a reliable means to detect S deficiency under field conditions. Measurement of this ratio by XRF presents the advantage of requiring only one measurement to be performed on grounded dry matter. Its use as a diagnostic tool of plant S nutrition requires accurate threshold values to adjust S fertilization accordingly. A determination method for these threshold values was suggested but will need to be tested at a larger scale (larger number of plots, adaptation to other cultivated species). Finally, the S fertilizer recommendation derived from this indicator will have to be verified a posteriori on yield measurement and/or seed quality.

## Figures and Tables

**Table 1:** SO_4_^2−^contents (mg g^−1^ DW), (Cl^−^+NO_3_^−^+PO_4_^3−^) contents (mg g^−1^ DW) and ([Cl^−^]+[NO_3_^−^]+[PO_4_^3−^]):[SO_4_^2−^] ratios in leaves of *B. napus*, *B. oleracea*, *T. aestivum*, *Z. mays*, *S. lycopersicum*,and *M. truncatula* following different S treatments (+S: control treatment, white columns, -S: S deprivation treatment, grey columns) of plants grown under controlled conditions (given in Supplemental Data SD2). Data are given as the mean ± SE (*n*= 4). *, ** and *** indicate significant difference between control and S deprived plants for *p* < 0.05, *p* < 0.01 and *p*< 0.001, respectively.

Species	Day of Treatment	SO_4_^2−^ Content (mg g^−1^ DW)		(Cl^−^+NO_3_^−^+PO_4_^2−^) Content (mg g^−1^ DW)		([Cl^−^]+[NO_3_^−^]+[PO_4_^2−^])/[SO_4_^2−^] Ratio	
		+S	−S	+S	−S	+S	−S
***B. napus***	0	24.38 ± 1.83		59.79 ± 7.37		2.32 ± 0.28	
	3	26.20 ± 1.23	15.90 ± 1.44***	63.65 ± 0.96	70.86 ± 5.68	2.43 ± 0.11	4.46 ± 0.39**
	13	29.85 ± 0.50	8.05 ± 0.48***	62.83 ± 5.86	86.42 ± 4.66**	2.10 ± 0.20	10.74 ± 0.17***
***B. oleracea***	135	7.05 ± 1.22	0.35 ± 0.04***	44.9 ± 6.85	61.87 ± 3.27*	6.36 ± 1.06	176.77 ± 40.89**
***T. aestivum***	0	3.03 ± 0.09		63.58 ± 0.80		20.98 ± 0.76	
	8	1.94 ± 0.05	1.15 ± 0.03	59.34 ± 2.10	68.04 ± 0.84**	30.58 ± 0.53	59.16 ± 2.36***
	16	2.17 ± 0.06	0.50 ± 0.05***	64.79 ± 1.29	70.19 ± 1.80*	29.85 ± 0.53	140.38 ± 15.81***
***Z. mays***	0	2.02 ± 0.49		74.74 ± 1.55		37.00 ± 7.16	
	5	3.44 ± 0.07	1.39 ± 0.08***	78.31 ± 2.82	91.71 ± 1.28**	22.76 ± 1.09	56.33 ± 3.45***
	18	1.38 ± 0.05	0.27 ± 0.02***	70.38 ± 3.46	103.28 ± 0.79***	51.00 ± 2.19	382.51 ± 23.44***
***S. lycopersicum***	75	63.75 ± 4.79	50.39 ± 2.42*	68.84 ± 4.61	87.77 ± 2.44*	1.07 ± 0.15	1.74 ± 0.10**
***M. truncatula***	0	3.31 ± 0.16		25.32 ± 0.60		7.55 ± 0.21	
	8	3.60 ± 0.12	0.15 ± 0.01***	25.40 ± 1.54	34.27 ± 3.44*	7.05 ± 0.48	228.47 ± 25.21***
	21	4.75 ± 0.18	0.26 ± 0.01***	25.92 ± 1.94	34.90 ± 2.46*	5.49 ± 0.50	134.23 ± 12.48***

**Table 2:** Soil type, soil S content (mg S kg^−1^), S and N fertilization managed by the farmers (kg S or N ha^−1^) and the value of the ([Cl]+[P]):[S] ratio in mature leaves harvested before or after fertilization in four oilseed rape commercial varieties from field experiment 2 (crops # 36′, 20′, 11′, and 8′). Status of plots have been determined within three groups (S deficient, at risk of S deficiency and S sufficient) according to threshold values of the ([Cl]+[P]):[S] ratio. Data are given as mean ± SE (*n* = 3). *, ** and *** indicate significant difference before and after fertilization for *p* < 0.05, *p* < 0.01 and *p* < 0.001, respectively. Letters when different indicate significant differences between crops at a given date, at *p* < 0.05.

			Before Flowering and Fertilization		S-(N) Fertilization (kg S-(N) ha^−1^	After Flowering and Fertilization	
**Plots number**	Soil type	Soil S content (mg kg^−1^)	([Cl]+[P])/[S] ratio	Status of plots		([Cl]+[P])/[S] ratio	Status of plots
**36′**	Compact silt	136	3.72 ± 0.43 b	S deficient	10-(166)	1.54 ± 0.21 b**	S deficient
**20′**	Superficial clay-limestone	239	2.64 ± 0.13 c	Risk of S deficiency	100-(146)	0.59 ± 0.10 a***	S sufficient
**11′**	Superficial clay-limestone	350	1.99 ± 0.14 a	S sufficient	0-(110)	1.42 ± 0.05 b**	S deficient
**8′**	deep clay-limestone	668	1.80 ± 0.06 a	S sufficient	15-(151)	1.41 ± 0.24 b	S deficient

## Supplementary Materials

The following are available online at http://www.mdpi.com/2223-7747/7/2/37/s1, Location of 45 commercial plots in France of field experiment 2; Multispecies experiment under controlled conditions; Composition of the two nutrient solutions used for control and S deprivation during the treatment period of *B. napus and Z. mays*.

## Abbreviations

DW	dry weight
HPLC	high performance liquid chromatography
HR ICP-MS	high resolution inductively coupled plasma mass spectrometry
XRF	X-ray fluorescence
