# Supplementary material for: Assessment of Sulfur Deficiency under Field Conditions by Single Measurements of Sulfur, Chloride and Phosphorus in Mature Leaves
Source: Plants (Basel). 2018 Apr 28;7(2):37. doi: 10.3390/plants7020037 (PMC6027431; doi:10.3390/plants7020037)
Supplement: Supplementary file 1 [file plants-07-00037-s001.pdf]

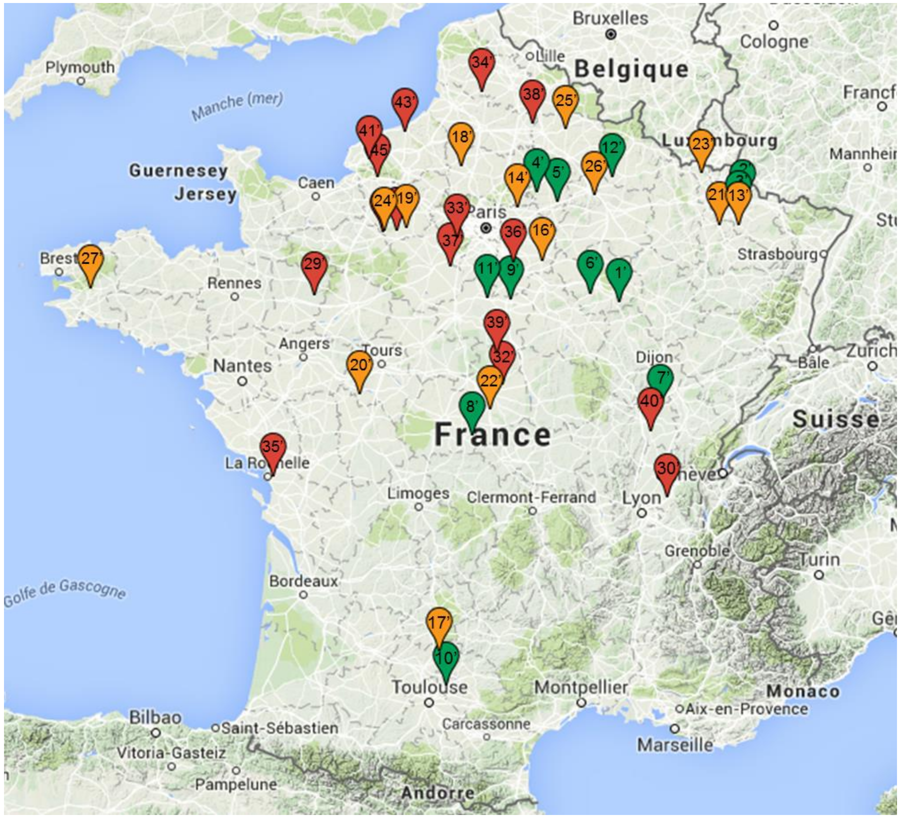

Supplemental data SD1 Location of 45 commercial crops in France of field experiment 2 with crops classified into three S status groups: S deficient in red, at risk of S deficiency in orange and S sufficient in green.

Supplemental data SD2: Multispecies experiment under controlled conditions.

B. napus and *Z. mays*

Seeds of *B. napus* L. cv Boheme and *Z. mays* cv Ronaldinio were germinated on perlite over demineralized water for seven days in the dark and then five days under natural light. Just after first leaf emergence, seedlings were transferred to hydroponic conditions (18 seedlings per 20L-plastic tank) in a greenhouse, between October and December for *B. napus* and between March and April for *Z. mays*, with a thermoperiod of 20°C (day) and 15°C (night). Natural light was supplemented with high-pressure sodium lamps (Master Greenpower T400W, Philips, Amsterdam, Netherlands) (350 µmol m^{-2} s^{-1} of photosynthetically active radiation at the canopy height) for 16h. The aerated nutrient solution contained: 3.75 mM KNO_3, 0.5 mM $MgSO_4$, 0.5 mM $CaCl_2$, 0.25 mM KH_2PO_4, 0.2 mM EDTA-2NaFe, 14 µM H_3BO_3, 5 µM $MnSO_4$, 3 µM $ZnSO_4$, 0.7 µM $CuSO_4$, 0.7 µM $(NH_4)_6Mo_7O_{24}$, 0.1 µM $CoCl_2$, 0.04 µM $NiCl_2$ and was buffered to pH 6.6 with 0.91 mM $CaCO_3$. This solution was renewed according to the rate of NO_3^- depletion monitored daily by using NO_3^- test strips (Merck Millipore, Darmstadt, Germany) in order to maintain optimal nutrition conditions. After four weeks of growth for *B. napus* and 8 days for *Z. mays*, plants were separated into two batches supplied with a modified nutrient solution chosen in order to achieve S deficiency and to maintain the same concentration of other nutrients (Supplemental data SD3): (i) control plants were grown with 508.7 µM SO_4^{2-}, (ii) S limited plants were grown with 8.7 µM SO_4^{2}. Nutrient solutions were renewed also according to NO_3^- depletion by monitoring the NO_3^- level in the tank.

T. aestivum

Seeds of *T. aestivum* L. cv Sankara were germinated on perlite over demineralized water for five days in the dark and then five days under light. Seedlings were transferred to hydroponic conditions (30 seedlings per 7L-plastic tank) in a growth chamber, with a thermoperiod of 22°C (day) and 18°C (night). Plants received artificial light provided by neon lamps (Lumilux cool daylight, 36W, Osram, Munich, Germany) (100 µmol m^{-2} s^{-1} of photosynthetically active radiation at the canopy height) for 12h. The aerated nutrient solution contained: 2 mM KNO_3, 1 $Ca(NO_3)_2$, 0.5 mM $MgSO_4$, 1 mM KH_2PO_4, 0.1 mM EDTA-2NaFe, 23 µM H_3BO_3, 5 µM $MnSO_4$, 2 µM $ZnSO_4$, 0.9 µM $CuSO_4$, 0.3 µM $(NH_4)_6Mo_7O_{24}$, 0.1 µM $CoCl_2$ and was buffered to pH 6 with

KOH. This solution was renewed every two or three days. After 11 days of growth, plants were separated into two batches: (i) control plants were grown with 507.9 µM SO_4^{2-}, (ii) S limited plants were grown with 7.9 µM SO_4^{2-}, replacing $MgSO_4$ by $MgCl_2$.

B. oleracea

B. oleracea plants cv Nikolas were grown individually in pots filled with a mixture of perlite: vermiculite (v:v, 1:1) in 1L pots for one month then in 2L pots for one month and finally in 8L pots for four months. The first part of the growth period, plants were grown in a growth chamber, with temperatures of 20°C during the day and 18°C during the night, a 14h photoperiod and a mean photosynthetically active radiation of 100 µmol m^{-2} s^{-1} guaranteed by the use of neon lamps (Lumilux soft white, L58W/840, Osram, Munich, Germany). Then plants were vernalized for two weeks at 10°C with a photoperiod of 12h. During the second part of the growth period, plants were transferred to a greenhouse with natural light and temperature controlled at 18°C during the day and 10°C during the night. Plants were watered throughout the experiment, every two days in the growth chamber and every day in the greenhouse, with a nutrient solution composed of 4.5 mM KNO_3, 3.6 $Ca(NO_3)_2$, 1.4 NH_4NO_3, 0.3 mM $MgSO_4$, 0.1 mM $MgCl_2$, 1 mM KH_2PO_4, 35 mg l^{-1} Fe-EDTA (FerVeg E13, Angibaud et spécialité, La Rochelle, France) and 3.5 mg l^{-1} OligoMix (Oligoveg S2, Angibaud et spécialité, La Rochelle, France). For S deficiency treatment the same solution described above was used except that $MgSO_4$ was removed and $MgCl_2$ was adjusted to 0.5 mM.

S. lycopersicum

S. lycopersicum cv Plaisance s/ Emperador was grown in a greenhouse between July and December under natural light temperatures controlled to 20°C during the day and 15°C during the night. Two plants were cultivated per rockwool segment (Grotop, Grodan, Roermond, Netherlands) and were watered every day by a nutrient solution containing: 1.3 mM KNO_3, 4.1 $Ca(NO_3)_2$, 1.7 NH_4NO_3, 1.9 mM $K_2(SO_4)$, 1.8 mM KH_2PO_4, 2.2 mM $Mg(NO_3)_2$, 1.1 mM $CaCl_2$, 15 mg l^{-1} Fe-EDTA (FerVeg E13, Angibaud et spécialité, La Rochelle, France) and 2.5 mg l^{-1} OligoMix (Oligoveg S2, Angibaud et spécialité, La Rochelle, France). For S deficiency treatment $K_2(SO_4)$ was reduced to 0.2 mM and KNO_3 was adjusted to 4.7 mM.

M. truncatula

M. truncatula plants (Gaertn. A17 genotype) were grown in a randomized complete-block design in a greenhouse temperature controlled to 20°C during the day and 18°C during the night. Artificial lighting was used to ensure 16h light per day: 400 W sodium lamps, three lamps in 17.32 m^2, radiation in the range 400-700 nm, and photosynthetic characteristics of 695 µmol s^{-1}. A number of mature seeds exceeding about twice the number of plants needed were scarified, imbibed with water for one day at room temperature, and then vernalized four days at 5°C. After vernalization, seeds were germinated at room temperature for one day and then placed on a small float raft system in the greenhouse. The germinated seeds were then set into small holes cut into styrofoam, with their radicle growing into the nutrient solution described in Zuber et al. (2013) containing 4 mM KNO_3, 4 mM $Ca(NO_3)_2$, 0.3 mM $MgSO_4$, 0.9 mM $MgCl_2$, 0.2 mM NaCl, 0.72 µM Na_2MoO_4, 0.10 mM EDTA-2NaFe, 8.2 µM $MnCl_2$, 1 µM $CuCl_2$, 1 µM $ZnCl_2$, 30 µM H_3BO_3, and 1 mM K_2HPO_4 (pH adjusted to 6.3 using H_3PO_4 before addition of K_2HPO_4). Homogeneous plantlets with two well-formed trifoliate leaves were then individually grown in 3L buckets under hydroponic conditions with vigorous aeration in the solution described above. For applying S deficiency (at a mid-vegetative stage characterized by the appearance of tertiary branches, the S1 stage in Zuber et al. 2013), the pots were rinsed with deionized water and then filled with the solution described above except that SO_4^{2-} was replaced by 1.16 mM $MgCl_2$.

Supplemental data SD3: Composition of the two nutrient solutions used for control and S deprivation during the treatment period of *B. napus and Z. mays*. Nutrient concentrations are expressed in mM.

Nutrients	Control	S deprivation
KNO_3	3.75	3.75
KH_2PO_4	0.25	0.25
EDTA, 2NaFe	0.20	0.20
$MgSO_4$	0.50	0
$CaCl_2$	0.50	0
$MgCl_2$	0	0.50
$CaCO_3$	0.91	1.41
H_3BO_3	1.4×10^{-2}	1.4×10^{-2}
$MnSO_4$	5×10^{-3}	5×10^{-3}
$ZnSO_4$	3×10^{-3}	3×10^{-3}
$CuSO_4$	7×10^{-4}	7×10^{-4}
$(NH_4)_6Mo_7O_{24}$	7×10^{-4}	7×10^{-4}
$CoCl_2$	1×10^{-4}	1×10^{-4}
$NiCl_2$	4×10^{-5}	4×10^{-5}
